# Haplotype-Based Genome-Wide Association Analysis Using Exome Capture Assay and Digital Phenotyping Identifies Genetic Loci Underlying Salt Tolerance Mechanisms in Wheat

**DOI:** 10.3390/plants12122367

**Published:** 2023-06-19

**Authors:** Raj K. Pasam, Surya Kant, Emily Thoday-Kennedy, M. Adam Dimech, Sameer Joshi, Gabriel Keeble-Gagnere, Kerrie Forrest, Josquin Tibbits, Matthew Hayden

**Affiliations:** 1 Agriculture Victoria, AgriBio, Centre for AgriBioscience, Bundoora, VIC 3083, Australia; raj.pasam@agriculture.vic.gov.au (R.K.P.); adam.dimech@agriculture.vic.gov.au (A.M.D.); kerrie.forrest@agriculture.vic.gov.au (K.F.); matthew.hayden@agriculture.vic.gov.au (M.H.); 2 Agriculture Victoria, Grains Innovation Park, Horsham, VIC 3400, Australia; emily.thoday-kennedy@agriculture.vic.gov.au (E.T.-K.);; 3 School of Applied Systems Biology, La Trobe University, Bundoora, VIC 3083, Australia

**Keywords:** high-throughput phenotyping, QTL, genome-wide association studies, salinity tolerance, wheat accessions

## Abstract

Soil salinity can impose substantial stress on plant growth and cause significant yield losses. Crop varieties tolerant to salinity stress are needed to sustain yields in saline soils. This requires effective genotyping and phenotyping of germplasm pools to identify novel genes and QTL conferring salt tolerance that can be utilised in crop breeding schemes. We investigated a globally diverse collection of 580 wheat accessions for their growth response to salinity using automated digital phenotyping performed under controlled environmental conditions. The results show that digitally collected plant traits, including digital shoot growth rate and digital senescence rate, can be used as proxy traits for selecting salinity-tolerant accessions. A haplotype-based genome-wide association study was conducted using 58,502 linkage disequilibrium-based haplotype blocks derived from 883,300 genome-wide SNPs and identified 95 QTL for salinity tolerance component traits, of which 54 were novel and 41 overlapped with previously reported QTL. Gene ontology analysis identified a suite of candidate genes for salinity tolerance, some of which are already known to play a role in stress tolerance in other plant species. This study identified wheat accessions that utilise different tolerance mechanisms and which can be used in future studies to investigate the genetic and genic basis of salinity tolerance. Our results suggest salinity tolerance has not arisen from or been bred into accessions from specific regions or groups. Rather, they suggest salinity tolerance is widespread, with small-effect genetic variants contributing to different levels of tolerance in diverse, locally adapted germplasm.

## 1. Introduction

Salinity stress threatens global food security, causing annual yield losses of up to 50% in salt-affected agriculture regions [1]. Salt-affected soils, including sodic and saline soils, are characterised by the presence of soluble salts, or their ions, at levels that are toxic to plant growth. Sodium chloride (NaCl), the most soluble salt, is widespread among saline soils and is commonly used to screen for plant salinity tolerance [2,3]. Soil salinity adversely affects crop germination, vigour, and yield through impacts on plant metabolism, photosynthesis, osmotic stress, ion toxicity, and nutritional imbalance. Prior research on plant responses to soil salinity has identified numerous adaptive mechanisms in different species [4,5,6]. Wheat is a major food crop and is grown globally across a broad range of climatic zones and soil types. Expanding soil salinity coupled with arid climates is a major problem for many wheat-growing regions, including Australia, South Asia, the Mediterranean basin, and Eurasian regions [7,8]. Although wheat typically has moderate salt tolerance, it is adversely affected at concentrations above 100 mM NaCl [5].

Salinity tolerance in wheat is a complex phenomenon involving various physiological, biochemical, and metabolic processes [9,10,11,12,13]. Major mechanisms for salinity tolerance in wheat, as well as other crops, are ion exclusion and shoot ion-independent tissue tolerance [14,15]. Early research in wheat focused on ion exclusion mechanisms that prevent the transfer of Na^+^ to the shoot. This research identified several large-effect transporter genes, which are now utilised in wheat breeding [16,17]. The sodium transporter genes *Nax1* and *Nax2*, inherited from *Triticum monoccocum* and mapped to the long arms of chromosomes 2A and 5A, respectively, impart salinity tolerance in wheat through the exclusion of Na^+^ from the xylem in roots [18,19]. The *Kna1* gene identified in wheat on the distal arm of chromosome 4D causes Na^+^ exclusion from leaves [20]. Tissue tolerance mechanisms typically involve Na^+^ sequestration and balancing of K^+^ uptake and retention in young shoots, while cytosolic exclusion of Na^+^ involves its transfer from young to older tissues or sequestration to vacuoles employing Na^+^/H^+^ antiporters or efficient intracellular compartmentalization [21,22]. Other adaptive mechanisms, such as leaf cell wall thickening, exclusion of toxic reactive oxygen species (ROS), salt stress signalling, and increased growth to dilute Na^+^ accumulation have also been proposed to help combat soil salinity in various crops [23,24]. In cereals, ion exclusion mechanisms have predominantly been studied due to the complexity of measuring salt response traits associated with other mechanisms [17,21].

Salinity tolerance can be assessed by measuring a range of salt response traits, including plant vigour, shoot biomass, shoot Na^+^ concentration, Na^+^ compartmentalization in older leaves, shoot K^+^/Na^+^ balance, and yield impacts measured under field or controlled environment conditions [12,25]. Field screening for salt tolerance is an arduous task as soil heterogeneity and the confounding effects of climatic and environmental factors, such as rainfall, temperature, light intensity, and humidity, can manifest as large genotype effects that are hard to control [26,27]. Consequently, screening in controlled environmental conditions is a preferred method of salinity tolerance assessment for large populations [25,28,29].

Digital phenotyping makes it possible to readily obtain high-accuracy, repeated, and non-destructive measures for various plant salinity response traits across large numbers of samples and time points. Trait examples include the rate of biomass accumulation, senescence rate, and various plant stress-related indices [30,31]. When deployed with traditional methods for measuring plant Na^+^ and K^+^ accumulation, digital phenotyping technologies can enable a thorough investigation of the prevalence and importance of different salinity tolerance mechanisms [9,28].

Genetic analyses based on bi- or multi-parental mapping populations and genome-wide association studies (GWAS) are widely used to investigate the genetic and genic basis for trait expression [32,33,34]. GWAS approaches using diverse germplasm have been shown to be advantageous over mapping populations, typically resulting in the mapping of QTL with higher resolution [35]. Several approaches are now being developed for GWAS based on single- and multi-locus SNP analysis [36,37]. Multi-locus approaches overcome many of the limitations of single-locus tests and have been used to investigate complex traits in plants and animals [36,38]. More recently, haplotype-based GWAS (HGWAS), in which SNP-based haplotypes are tested for trait association, has been developed and shown to increase statistical power for QTL detection [39,40,41]. SNP haplotypes for HGWAS can be constructed from gene coordinates and fixed physical intervals in sequenced genomes or based on linkage disequilibrium (LD) [40].

Wheat improvement against salinity requires approaches that include different ion exclusion, sequestration, and osmotic tolerance mechanisms to efficiently surmount the limitations imposed by soil salinity. Apart from the complexity of the trait, the large genome size of bread wheat (~16 Gb) [42] imposes considerable challenges in mapping loci for salt response. The influx of affordable genotyping technologies now allows the genotyping of large numbers of wheat accessions with dense marker data using SNP chips [43,44], DArT technology [45] genotyping-by-sequencing assays [46], and exome capture sequencing [47]. With the availability of whole genome marker data and advances in statistical genomics, GWAS and genomic selection (GS) approaches are being widely used to understand the genetic landscape of various complex traits in wheat [33,48,49] and other major crops [50,51,52]. Understanding salinity tolerance mechanisms and the genetic basis for these mechanisms is important for wheat improvement against salinity. Deciphering the genetics underlying such complex traits requires screening large numbers of accessions. The aim of this study was to characterise genetic variation for salinity tolerance in globally diverse cultivated wheat germplasm. Here, automated digital phenotyping was implemented to establish growth and stress-related traits to efficiently screen a large wheat population. Combining this with HGWAS analysis allowed known and novel QTL to be identified for salinity tolerance. This study provides a strong base to link advanced phenotyping and genotyping analytics to elucidate the genetic basis of salinity tolerance in wheat.

## 2. Results

To investigate the genetic basis for salinity tolerance, we applied a high-throughput phenotyping assay, previously demonstrated to be discriminatory of salt tolerance and correlated to field responses [25,28], to screen globally diverse wheat accessions (Supplementary Table S1). A total of 33 traits, including both measured and derived traits, were used to evaluate vegetative growth stage salinity response in two separate experiments conducted at two glasshouse phenotyping facilities, Plant Phenomics Victoria, Bundoora (PPV-B) and Horsham (PPV-H), located in Victoria, Australia. Traits were analysed separately by facility; those with the suffix ‘B_’ and ‘H_’ were measured in the PPV-B and PPV-H, respectively (Supplementary Table S2).

### 2.1. Estimation of Digital Shoot Growth Rate (dSGR) and Its Variation in Wheat

**Figure 1:**
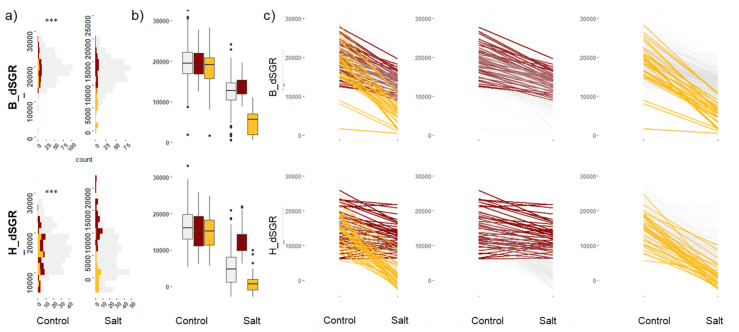
Phenotype variation observed for digital shoot growth rate. The top panel of plots corresponds to the digital shoot growth rate (dSGR) measured in the PPV-B, and the bottom panel of plots corresponds to the PPV-H. dSGR values are represented on the *y*-axis for all plots. The *p*-value for the test of significant difference between means of control and salt treatments based on ANOVA is shown on the top left of the figure (*** denotes *p*-value < 0.0001). Colour code based on SSI: red, tolerant in both locations; yellow, susceptible in both locations; grey, moderately tolerant to susceptible. (**a**) Histogram showing distribution of shoot growth rate under both control and salt-treated conditions. (**b**) Boxplots showing the phenotype variation. (**c**) Reaction-norm plots for shoot growth rate between control and salt-treated plants. The left-most plot shows all accessions; the middle plot Tolerant accessions and right-plot susceptible accessions.

Dry biomass was measured by a destructive manual method at the end of experiments performed in the PPV-H to establish the relationship between dry biomass (DB) and digital shoot area extracted from images. Dry biomass estimates were highly correlated (r > 0.9) for digital shoot area in both control and salt-treated plants (Supplementary Figure S1). The digital shoot area was used as a proxy for biomass at each timepoint to calculate the digital shoot growth rate (dSGR) and rate of biomass accumulation over time (see methods; Supplementary Figure S2). Digital shoot growth rate values correspond to the accumulated pixel area per day during the course of the experiment. Extensive phenotypic variation was observed for dSGR, with values ranging from 1694 to 33,071 in control plants and from 0 to 24,139 in salt-treated plants (Figure 1). The overall mean for dSGR in control and salt treatment was 19,534.43 pixels and 12,146.80 pixels and 16,230.85 pixels and 5593.67 pixels for the PPV-B and PPV-H experiments, respectively. The percentage loss of dSGR under salt treatment for individual plants was between 2% and 100%, with dead plants considered a 100% loss.

### 2.2. Salt Susceptibility Index (SSI) to Determine Tolerance

Values of dSGR from plants under control and salt treatment were used to calculate SSI. dSGR_SSI was used to assign accessions that were phenotyped at both locations (*n* = 372) into three broad salinity tolerance groups: tolerant in both locations (*n* = 44), susceptible in both locations (*n* = 33), and the rest of the accessions (Supplementary Table S1). Values for dSGR_SSI ranged from 0 to 2.5, where the value 0 indicated no difference in dSGR between the control and salt-treated plants, and higher values indicated increasing loss of shoot growth under salt treatment. Accessions with SSI values < 1.0 were considered tolerant, and those with SSI values >1.5 were considered susceptible.

### 2.3. Leaf Elemental Analysis

**Figure 2:**
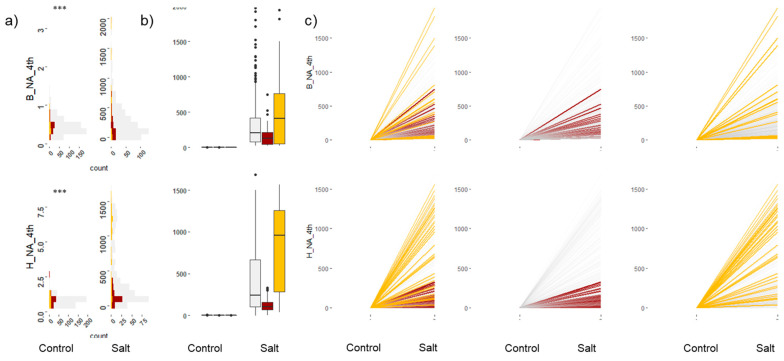
Sodium (Na^+^) accumulation in the oldest shoot tissue, fourth leaf from bottom. The top panel of plots corresponds to Na^+^ in the PPV-B, and the bottom panel of plots corresponds to Na^+^ in the PPV-H. The *y*-axis values correspond to Na^+^ accumulation in the fourth leaf. The *p*-value for the test of significant difference between means of control and salt treatments based on ANOVA is shown on the top left of the figure (*** denotes *p*-value < 0.0001). Colour code: red, tolerant in both locations; yellow, susceptible in both locations; grey, moderately tolerant to susceptible. (**a**) Histogram showing distribution of Na^+^ concentrations in leaf tissue under both control and salt-treated conditions. (**b**) Boxplots showing the phenotype variation. (**c**) Reaction-norm plots for Na^+^ between control and salt-treated plants.

Leaf Na^+^ and K^+^ concentrations were measured in the fourth leaf from the bottom and the second youngest leaf from the top of the main tiller. Significant differences were observed for leaf Na^+^ and K^+^ accumulation and for the K^+^/Na^+^ ratio between treatments and across accessions (Figure 2). Most tolerant accessions showed less Na^+^ accumulation (<100 µM Na^+^ g^−1^ DW) in the younger leaf (second leaf) at both locations. Interestingly, four accessions had Na^+^ concentrations of up to 350 µM Na^+^ g^−1^ DW and dSGR_SSI values close to one. For Na^+^ accumulation in the oldest leaf (fourth leaf), the most tolerant accessions had values < 300 µM Na^+^ g^−1^ DW. Most susceptible accessions had high Na^+^ accumulation in both leaves, although a few had low Na^+^ accumulation (Figure 2 and Figure S3).

**Figure 3:**
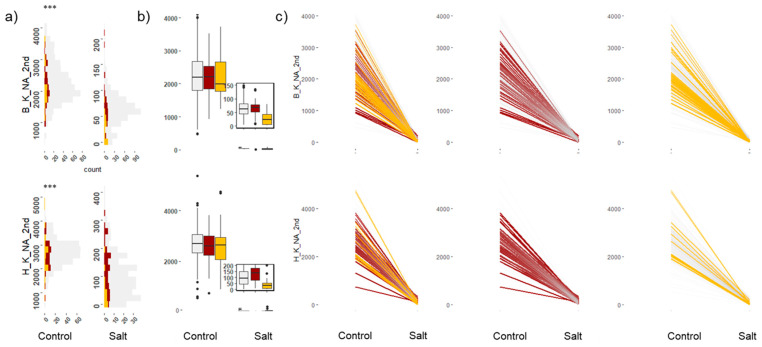
Potassium to sodium (K^+^/Na^+^) ratio in the youngest shoot tissue, second leaf from top. The top panel of plots corresponds to K^+^/Na^+^ in the PPV-B, and the bottom panel of plots corresponds to the PPV-H. The *y*-axis values correspond to the ratio of K^+^/Na^+^ in the second leaf. The *p*-value for the test of significant difference between means of control and salt treatments based on ANOVA is shown on the top left of the figure (*** denotes *p*-value < 0.0001). Colour code: red, tolerant in both locations; yellow, susceptible in both locations; grey, moderately tolerant to susceptible. (**a**) Histogram showing distribution of K^+^/Na^+^ ratios in leaf tissue under both control and salt-treated conditions. (**b**) Boxplots showing the phenotype variation. (**c**) Reaction-norm plots for K^+^/Na^+^ between control and salt-treated plants.

K^+^ concentrations in the young leaf were reduced under salt treatment, while the oldest leaves showed higher reductions. Accessions in the tolerant group had the highest K^+^ levels (Supplementary Figures S4 and S5) in both leaves under salt treatment. The K^+^/Na^+^ ratio was also reduced in both leaves, with the tolerant group accessions having the lowest reduction in ratio (Figure 3). Most accessions in the tolerant group had K^+^/Na^+^ ratio values > 50 in the young leaves, while accessions in the susceptible group had values < 20 (Supplementary Figure S6).

**Figure 4:**
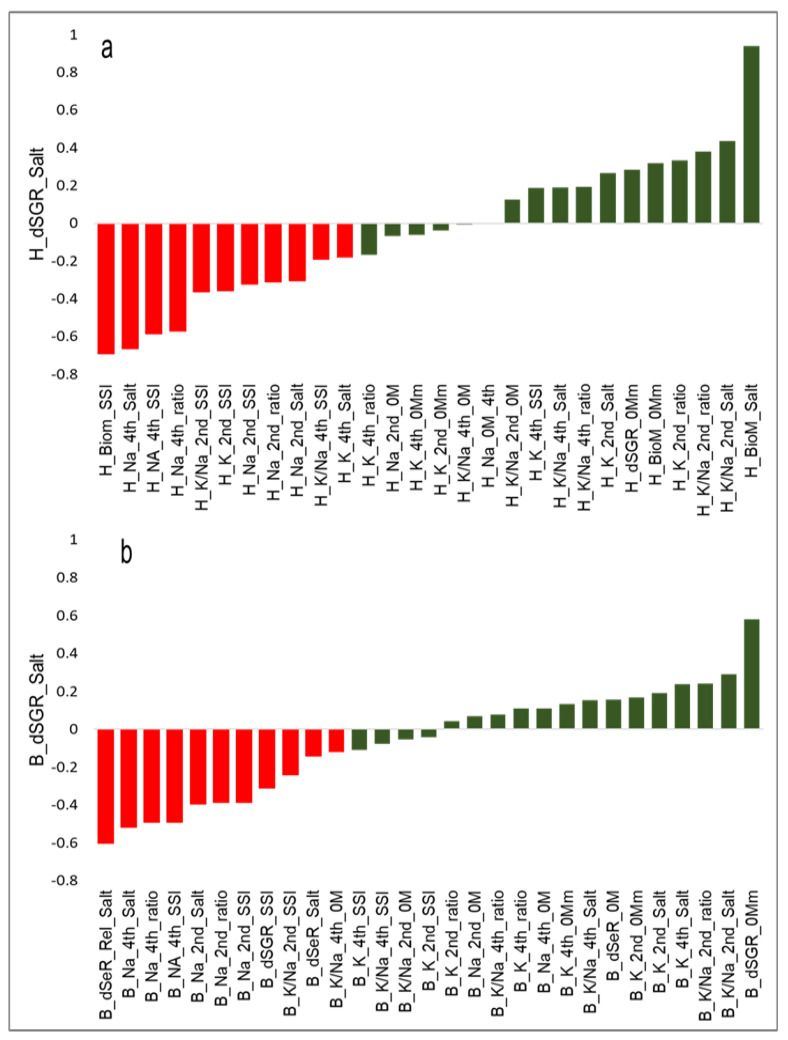
Correlation between growth rate (dSGR) under salt treatment and other measured traits at the (**a**) PPV−H and (**b**) PPV−B. Positive and negative correlations (r^2^) are coloured green and red, respectively, and the trait names correspond to the trait description provided in Supplementary Table S2. *Y*-axis values correspond to correlation coefficients, and *x*-axis values represent trait names.

Correlations among the leaf elemental traits measured in both PPV-H and PPV-B showed that dSGR under salt treatment was most strongly positively correlated with the K^+^/Na^+^ ratio in the youngest leaf (H_KNa_2nd_Salt) and most strongly negatively correlated with Na^+^ accumulation in the oldest leaf (H_Na_4th_Salt; Figure 4).

### 2.4. Haplotype Analysis

Across the genome, 58,502 haplotype blocks were detected, with 22,320, 31,055, and 5127 hapblocks on the A, B, and D genomes, respectively (Supplementary Figure S7). The hapblocks ranged from 2 bp to 1 MB in size and had an average size of 116 kB, with 2 to 341 SNP per block. Most hapblocks (74%) had <15 SNP, and 613 hapblocks had >100 SNP. After removing hapalleles with a <0.01 minor allele frequency (MAF), 266,760 hapalleles remained. On average, each hapblock had three hapalleles, with a range of two to twenty-two hapalleles. The number of hapalleles per block was proportional to the number of SNPs within the hapblock. Haplotype diversity varied across the genome, with the distal regions of the chromosomes having a larger number of hapalleles compared to the centromeric regions. The D-genome had the least number of hapalleles and hapblocks (Supplementary Figure S7).

### 2.5. Haplotype-Based Genome-Wide Association Analysis (HGWAS) and Genetic Correlations

**Figure 5:**
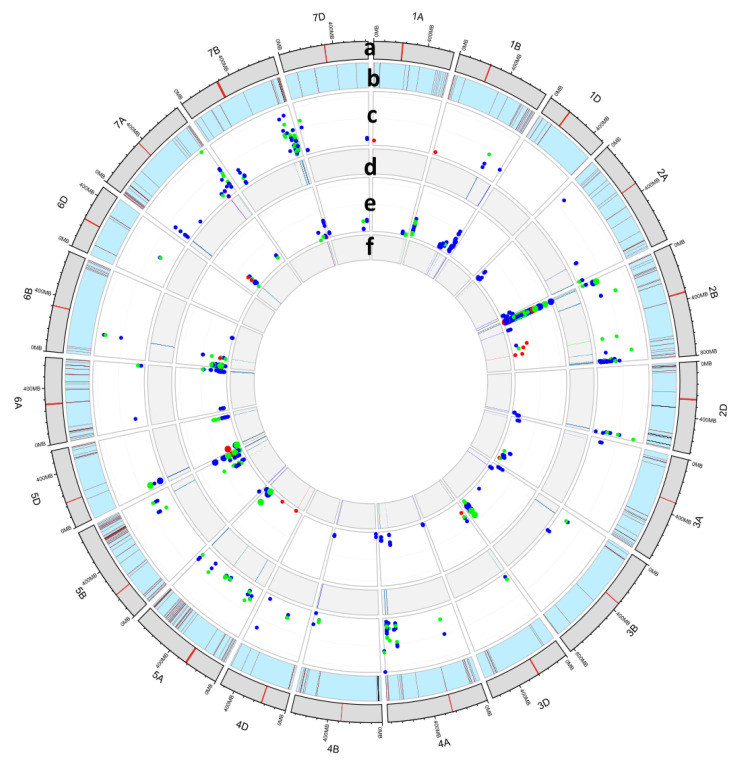
The genetic landscape of salinity tolerance in wheat showing 95 QTL detected by the HGWAS approach. Colour code: red, growth-related traits; green, leaf ion concentration; and blue, salt stress index (comparison of control to salt). Track-**a** corresponds to chromosomes and physical positions. Track-**b** in light blue shows the physical position of previously reported QTL, with positions having > 3 QTL shown in black and the remainder with brown bars. Track-**c** and Track-**e** show GWAS signal log_10_(P) values in the PPV-H and PPV-B environments. Only associations with log_10_(P) > 5.00 and that passed the CV threshold are plotted. Track-**d** and Track-**f** in grey show QTL intervals identified.

A five-fold cross-validation and *p*-value threshold were used to identify trait associations within hapblocks. Across all traits, a total of 662 haplotype-allele-trait associations were detected. These associations corresponded to 267 hapalleles from 236 hapblocks. Several of the hapblocks were associated with multiple traits in both the PPV-H and PPV-B environments (Supplementary Table S3). Hapallele associations within a 4 MB physical interval (or up to 8 MB when consecutive hapallele associations were present) were considered a single QTL region, which resulted in 95 trait associated QTL across 19 chromosomes. The largest number of QTL were detected on chromosome 2B (*n* = 11), followed by 7B (10), and 7A (9). No QTL were detected on chromosomes 1D and 6D (Figure 5 and Supplementary Table S3). Among the 662 hapallele trait associations, 567 were significant at the modified Bonferroni correction threshold. Hapallele trait associations (*n* = 95) that did not cross the FDR threshold corresponded to 48 hapblocks and 11 QTL. Here, we reported all hapallele trait associations that were significant in more than 50 cross-validation runs at *p*-values < 0.00001.

Classification of the estimated traits into three groups based on their functionality—growth-related, leaf salt ion, and salt stress index—showed that most of the QTL harboured associations for traits related to salt stress index (*n* = 81), followed by leaf ions (*n* = 64) and growth-related traits (*n* = 19). Seven QTL (Figure 5 and Supplementary Table S3) were associated with all three trait categories; 59 were associated with a two-trait category, and 29 were associated with a single-trait category. Haploallele-to-trait associations per QTL ranged from 1 to 97, with QTL.2B.24_27 showing the highest number of associations.

A few QTL had >10 associations for different traits and were considered important genomic regions. For example, the QTL in chromosome 2B between 24 and 27 MB contained 15 hapblocks associated with 11 traits with multiple hapallele associations (QTL.2B.24_27; Supplementary Table S3). Similarly, QTL with multiple trait associations for the three categories of traits were detected in chromosomes 3B, 5A, 3D, 5D, 6B, 7A, and 7B (Figure 5 and Supplementary Table S3). The QTL in chromosome 7B between 719 and 727 MB (QTL.7B.719_727) harboured 39 associations with six traits from three categories (indices, leaf salt ions, and growth-related). On the same chromosome, about 20 MB proximal to this QTL, another cluster of associations (QTL.7B.686, QTL.7B.693_694, and QTL.7B.699_701) with leaf ion concentration and salt tolerance traits were detected. Ten QTL were associated with growth-related trait susceptibility indices across chromosomes 1B, 2A, 2B, 4B, 4D, 5D, and 7A (QTL.1B.688_693, QTL.2A.67, QTL.2B.24_27, QTL.4B.660_664, QTL.4D.445, QTL.5D.44_49, QTL.6A.89, QTL.7A.642_644, QTL.7A.50_55; Figure 5 and Supplementary Table S3).

Genetic correlations between the traits are reported in Supplementary Figure S8. A high genetic correlation between B_dSGR_SSI and H_BioM_SSI (r_g_^2^ = 0.72) and a moderate correlation for dSGR_SSI between both locations (r_g_^2^ = 0.25) indicated the underlying genetic architecture for this trait was similar at both locations. High r_g_^2^ values were not observed for other traits between locations due to GxE variation for most of the salinity tolerance traits. Response to salinity is growth stage-specific, and low correlations have been reported for salinity tolerance traits measured in two locations for the same accessions [29]. Previous salinity tolerance field studies in wheat have reported large GxE effects between seasons at the same location [53]. In this study, low-to-moderate genetic correlations were observed for the ion accumulation traits between the locations, and for these traits, common QTL were detected at both locations (Supplementary Table S3 and Supplementary Figure S8).

### 2.6. Gene-Based Meta-Analysis and Gene Ontology Terms Enrichment

Based on the position of the 95 QTL in IWGSC RefSeq v2.0, gene coordinates and functional annotation information for 3134 genes located within 2MB of the QTL were used for gene-based meta-analysis (Supplementary Table S4). Of these genes, 11 were related to Na^+^ or K^+^ transport, and 182 were involved in transporter activity (Supplementary Table S4). As the gene-based test only considers genes with >1 polymorphic SNP within the gene and an associated 2 KB upstream and downstream region, nearly half of the genes (*n* = 1492) were excluded in the analysis. This included most of the genes with transporter functions. GWAS summary statistics for the genes and associated 2 KB upstream and downstream regions were used for FastBAT analysis to identify 107 genes with significant associations (*p* < 0.001) for different salinity tolerance traits (Supplementary Table S5). Significant gene associations were detected for 36 QTL in the meta-analysis with multiple trait associations (Supplementary Figure S9), which were prioritised as putative functional candidate genes.

**Figure 6:**
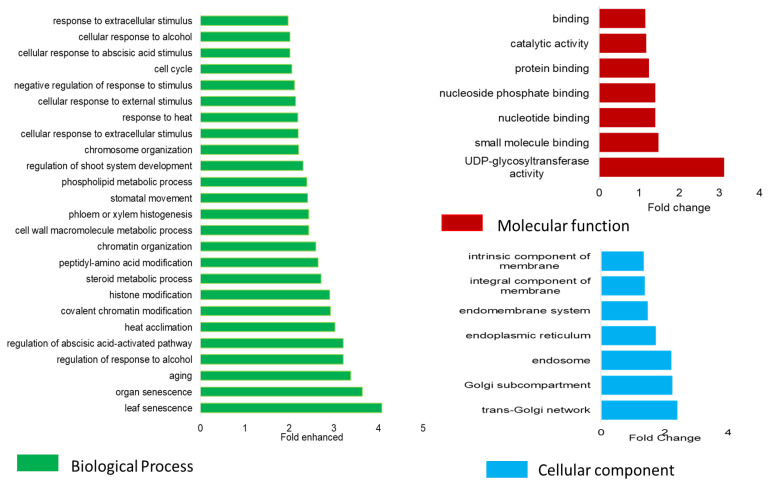
Gene ontology enrichment analysis with significant genes (*n* = 107) underlying the salinity tolerance QTL. Only the top 20% of GO terms with significant enrichment are plotted. Colour code: blue, cellular component; red, molecular function; and green, biological function.

Gene and gene ontology (GO) term enrichment analysis using the full list of genes (*n* = 3134) underlying the 95 QTL resulted in 2497 GO terms, of which 1764, 485, and 287 were related to biological processes, molecular functions, and cellular component functions. Dominant GO terms (*p*-value < 0.05) corresponding to biological processes (*n* = 263), cellular components (*n* = 15), and molecular function (*n* = 110) are reported in Supplementary Table S6, and the top 20% of these enriched terms are plotted in Supplementary Figure S10. Repeating GO enrichment analyses using the significant gene list (*n* = 107) revealed 435 GO terms corresponding to cellular components (*n* = 54), molecular function (*n* = 55), and biological processes (*n* = 326), of which 39 GO terms were significantly enriched (*p*-value < 0.05) in this gene set (Figure 6; Supplementary Table S7). Results from statistical overrepresentation of GO terms were also performed in PANTHER, and the results were compared for overlapping significantly enriched GO terms (Supplementary Tables S6 and S7).

## 3. Discussion

This study aimed to investigate the extent of genetic variation for salinity tolerance in diverse cultivated bread wheat germplasm. We applied a high-throughput salinity tolerance screening assay [9,28] incorporating automated digital imaging to phenotype a large worldwide collection of genotypically diverse wheat accessions. In addition, leaf elemental analysis was conducted to investigate the partitioning of Na^+^ and K^+^ ions in leaves and provide an understanding of the physiology and genetics of salinity tolerance in wheat. The phenotype data were combined with genome-wide exome capture genotype data [47] to investigate the genetic basis of salinity response using haplotype-based GWAS. QTL for component traits related to salinity tolerance were identified and their genic basis investigated using gene-based meta-analysis and gene enrichment analysis.

High-throughput salinity tolerance screening assays, coupled with reliable phenotyping methods, enable accurate screening of large numbers of accessions in a short period of time. Here, we used automated digital imaging to derive direct measures and surrogate traits, such as projected pixel area of green and non-green leaf area, to accurately estimate biomass, growth rate, and senescence. Such measures have been used previously to evaluate abiotic stress tolerance in crops (Hairmansis et al., 2014; Meng et al., 2017). In this study, a high correlation was observed between digital shoot area and directly measured shoot biomass under both control and stress conditions (r = 0.95), giving us confidence to use these surrogate measures. The dSGR under salt stress varied significantly from control conditions, with tolerant lines maintaining higher dSGR than susceptible lines under saline conditions. The salt susceptibility index calculated with dSGR in control and salt conditions was used as the determinant trait for salt tolerance since it provides a direct measure of salt response per se and can therefore be considered a proxy trait for growth stage salinity response.

Our results show that under salinity, dSGR was negatively correlated to the rate of senescence (r = −0.61), followed by Na^+^ concentration in old leaves (r = −0.52), and positively correlated with the K^+^/Na^+^ ratio in young leaves (r = 0.43). Significant but low correlations were observed for Na^+^ concentration in young leaves with dSGR under salt. Previous studies have reported both similar and contrasting relationships. For example, no significant relationship was found between Na^+^ and K^+^ concentrations in shoots and overall salinity tolerance [10,54], and moderate to high correlations have been reported between shoot Na^+^ concentration and salinity tolerance in wheat [26,29] and other plants [55]. Variability in correlations between these studies is likely due to their specific conditions, such as population size, growth stage, growing medium, and duration and level of salt stress imposed.

Investigation of trait interactions between accessions grouped as tolerant, moderately tolerant, and susceptible showed that most tolerant lines had relatively low concentrations of Na^+^ in young leaves and low-to-moderate Na^+^ concentrations in old leaves compared to the other accession groups. Tolerant lines that had low Na^+^ concentrations in both old and new leaves could have multiple tolerance mechanisms that prevented both Na^+^ uptake and movement to young shoots. A few tolerant lines showed high-to-moderate Na^+^ concentrations in old leaves and low Na^+^ concentrations in young leaves (Figure 2). In the susceptible group, most accessions had high Na^+^ concentrations in older leaves. Controlled uptake and transport of Na^+^ from root to shoot and limited Na^+^ flux to young shoots by sequestering it in vacuoles or limiting it to old leaves have been attributed as important salinity tolerance traits for maintaining growth [14,56]. In addition, shoot ion-independent tolerance mechanisms, which include signalling pathways, balancing ROS levels, ion homeostasis, osmotic adjustment, compatible solutes, and tissue damage control, are contributing traits for salt tolerance in wheat [57,58]. In barley, plants with multiple tolerance mechanisms were shown to be highly tolerant to salinity [57]. Given the difficulty of distinguishing tissue tolerance and Na^+^ exclusion mechanisms and quantifying their contribution to salinity tolerance, measures of biomass accumulation and extent of chlorosis or senescence, along with leaf ion measures, can be useful to predict tissue tolerance in genotypes [59].

Several studies have reported QTL for salinity tolerance in wheat and other crops using high-throughput imaging platforms with limited numbers of accessions [60,61]. Salt tolerance exhibited at the vegetative growth stage was shown to be predictive of salt tolerance at later stages [29,62]. The shoot biomass under salt stress was shown to closely relate to overall salinity tolerance [25,29]. In this study, significant hapallele associations with salinity tolerance component traits were delineated into 95 QTL. While some of the QTL were located adjacent to one another (<20 MB) and might correspond to the same locus, merging them into the same locus will require further evidence. Forty-one of the QTL were co-located with previously reported QTL for salinity tolerance detected at the seedling stage or in field screening (Supplementary Figure S9 and Supplementary Table S3) [9,10,12,13,63,64,65,66,67,68] based on the physical position of SSRs [69] and SNPs [70] used in the different studies. Of the remaining 54 QTL, most appeared novel, with none located within 10 MB of a previously reported QTL. The detection of novel QTL in our study likely reflects a combination of the high marker density, the size of the association mapping population, and the diversity of the panel, which was specifically chosen to capture global genetic and geographic diversity in wheat [47].

Nineteen QTL for growth-related traits (dSGR, biomass, and dSeR) were detected on chromosomes 1A, 1B, 2B, 3A, 3B, 3D, 5A, 5B, 5D, 6B, 7A, and 7B. Most were also associated with leaf ion concentrations and respective salt tolerance indices (Supplementary Table S3). Among them, 10 QTL co-localised within the vicinity of previously mapped QTL for salinity tolerance component traits, especially yield-related traits. For example, QTL.5A.113_115 associated with senescence and Na^+^ index traits co-located within a QTL reported for relative root dry biomass and germination percentage under salt [13,64], and QTL.1A.10_11 and QTL.7A.642_644 overlapped with spike and yield-related QTL detected in field studies [47,65]. Salinity was shown to drastically affect spike-related yield traits in susceptible genotypes [71]. Similarly, QTL.2A_67 associated with biomass and senescence traits overlapped with previously mapped salinity tolerance QTL detected at the seedling and germination stages [64].

Enrichment analysis of the full gene list (*n* = 3134; Supplementary Table S4) underlying the 95 QTL identified in this study found enrichment for GO terms corresponding to metabolic activities, catalytic activity, terpene biosynthesis pathways, oxidoreductase activity, transporter activity, and detoxification functions (Supplementary Figure S10). Plants tend to accumulate secondary metabolites and upregulate secondary metabolism in response to abiotic stress as an adaptation strategy. The accumulation of phenylproponoids and terpenoids under stress was shown to relate to stress tolerance in wheat [72] and maize [73]. Terpenoid derivatives, such as abscisic acid (ABA), gibberellic acid, and strigolactones, are involved in plant development and adaptation against a range of abiotic stresses [74]. The QTL regions also showed enrichment of genes related to functions involving ion transport, detoxification, oxido-reductases, and antioxidant biosynthesis activities.

A gene-based meta-analysis identified 107 genes across 36 QTL to be significantly associated with salinity traits. For the remaining QTL, it could be either that the genes did not contain enough SNPs for meta-analysis or none of the genes passed the gene-based *p*-value threshold (<0.00001). Enrichment analysis revealed GO terms related to senescence, ageing, organelle repair, detoxification, metabolite synthesis, heat acclimatisation, and metal ion transport functions (Figure 6 and Supplementary Table S6). Of the 77 genes contained within the QTL.2B.24_27 interval, only one was significant in the meta-analysis. This gene was TraesCS2B02G048100, which is functionally annotated as glyoxylate reductase (GR) and was associated with GO terms classified as oxidation-reduction process, cation transport, membrane components, and metabolic process. GR genes are suggested to be involved in redox balance, detoxification by reduction of glyoxylate and succinic semialdehyde, and simultaneously involved in the production of osmolytes, which can reduce the impact of stress on cellular functions [75]. Previous studies in durum wheat suggest that GR genes are involved in the production of protective metabolites that mitigate damage to cellular organelles in the presence of salt and other stress stimuli [76,77].

Another QTL on 5D (QTL.5D.44_49) was associated with senescence and Na^+^ ion accumulation traits and contained 54 underlying genes, 18 of which had significant associations in the gene-based meta-analysis (Supplementary Table S5). Of these genes, TraesCS5D02G047100 is functionally annotated as a β-1,3-galactosyl transferase-like protein; its orthologues in *Arabidopsis* are reported to be upregulated under salinity and play a role in senescence [78]. Galactosyltransferases are involved in microfilament organisation, cell wall biosynthesis, and endomembrane organisation, resulting in ROS balancing in cells [78,79], and were associated with a dominant GO term for leaf senescence in the GO enrichment analysis (GO:0010150; Supplementary Table S7). A gene encoding β-1,3-galactosyl transferase-like protein (TraesCS5D02G461600) was also present among the 10 significant genes found in the gene-based meta-analysis for QTL.5D.506_508 (Supplementary Table S3). Gene ontology enrichment analysis showed that GO:0010150, annotated as leaf senescence function, is enriched with up to six genes associated with this GO term (Supplementary Table S6). Among these six genes, TraesCS5D02G461600 in the QTL_5D_506_508 interval is significantly associated with the rate of senescence in gene-based meta-analysis. As several of the significant genes associated with QTL.5D.44_49 and QTL.5D.506_508 in the gene-based meta-analysis were functionally annotated with relevant stress tolerance activities, our results support the implementation of functional validation studies to confirm candidate gene function in contributing to salinity tolerance in wheat. More broadly, principal component analysis based on genotype data revealed no obvious clustering of susceptible and tolerant accessions (Supplementary Figure S11), indicating that tolerance is not associated specifically with a single gene or narrow genetic origin. No specific enrichment for salinity tolerance was observed in accessions from any geographical region or in cultivars or landrace groups.

Our findings suggest salinity tolerance does not arise from or has been bred into accessions from specific regions or groups. Rather, they suggest salinity tolerance is widespread, with genetic variants contributing to different levels of tolerance in diverse, locally adapted germplasm. This observation was further supported by investigating enrichment for salinity tolerance between the tolerant and susceptible grouped accessions using the 28 hapalleles associated with dSGR_SSI and its correlated traits, which found no significant enrichment of alleles in the resistant group. This finding indicates multiple small effect QTL in germplasm contribute to tolerance in different backgrounds. Consistent with previous studies, our results demonstrate the genetic architecture underlying salinity tolerance in wheat is complex and controlled by multiple small effect QTL [13,64]. Increasing population size and improved phenotyping will aid in the discovery of more QTL and accurately assess the effects of the QTL discovered in this study. Our results provide a list of new and confirmed QTL for salinity tolerance component traits and suggest that multiple mechanisms underlie tolerance in globally diverse wheat germplasm.

## 4. Materials and Methods

### 4.1. Plant Material

The association mapping panel (AMP) used to screen for salinity tolerance consisted of 580 genetically and geographically diverse wheat landrace accessions and cultivars (Supplementary Table S1) selected from a globally diverse wheat collection of about 6700 accessions [47]. The population structure and genetic diversity of these accessions are described in [47]. SNP genotype calls derived from the exome capture sequence reported in [47] for each accession were used for GWAS analysis.

### 4.2. Growth Conditions and Phenotyping Trait Measures

The AMP was screened at two locations in controlled-environment glasshouses, known as the Plant Phenomics Victoria, Bundoora (PPV-B) and Plant Phenomics Victoria, Horsham (PPV-H) facilities in Victoria, Australia. Both facilities are equipped with the Scanalyzer 3D plant-to-sensor high-throughput phenotyping system (LemnaTec GmbH, Germany). A detailed description of the facility is provided in our previous study [80]. Experiments conducted at the two phenotyping facilities were conducted as biological repeats.

Each wheat accession was sown in a white plastic pot (200 mm diameter × 190 mm deep; Garden City Plastics Pty Ltd., Victoria, Australia) filled with standard potting mix (Biogro, South Australia, Australia) and fertiliser to ensure optimal growth. Three seeds were sown per pot and thinned to one plant at the three-leaf stage, keeping the plant of consistent size across genotypes. The pots were kept on saucers throughout the experiment to prevent water/saline solution loss. The glasshouse was maintained at 24 °C during the day and at 15 °C during the night. At the four-leaf stage (Zadoks stage 14, Z14 [81], ~15 days after sowing), salinity treatment started, where 125 mM NaCl treatment was applied in three split doses of 41, 42, and 42 mM on three consecutive days to the salt-treated pots, while the control pots did not receive any salt treatment and were allowed to grow under optimal conditions. The saline solution was added until it was leaching from the bottom of the pot. Excess leached solution was discarded from the saucers to maintain a uniform solution inside the pot. Once the required salt level was reached, the pots were watered on alternate days to maintain a constant pot weight. At the PPV-B facility, all 580 AMP accessions were phenotyped, and at the PPV-H facility, a subset of 372 accessions were phenotyped.

The plants were imaged every second or third day on the Scanalyzer 3D system using high-resolution digital top and side-mounted RGB cameras (Prosilica GT 6600C, Allied Vision Technologies, Stadtroda, Germany) having 28.8 megapixels, 14-bit resolution, a frame rate of 4 fps, and a cell size of 5.5 µm. The camera mounted directly above the plants was used to acquire the top-view image. Two side-view images were captured after consecutive rotations of the plant at 0° and 90°. Images were processed using LemnaGrid software (Lemnatec GmBH, Aachen, Germany) to extract total plant pixel area and relative non-green pixel area (non-green area/total area) at each time point. The full-colour images were processed by separating the region of interest (i.e., the plant) from the background. Colour classes defined as green, chlorotic, and necrotic were used to classify green and non-green plant pixels. Details of the image analysis pipeline are described in [80]. Fresh and dry biomass weights were measured by harvesting the plant material when the plants reached Zadoks stage 24, Z24 (35–36 days after sowing). Whole leaf samples of the second youngest leaf from the top of the plant and the fourth oldest leaf from the bottom were collected from each of the control and salt-treated plants for each accession and assayed for total Na^+^ and K^+^ ion concentrations (described below). The 33 traits observed, derived measures collected, and ratios and indices calculated are described in Supplementary Table S2.

### 4.3. Phenotype Data Analysis

Data analysis for all phenotypic measures was performed using R software [82], unless otherwise specified. The best linear unbiased estimates (BLUEs) were calculated for each accession. Trait definitions and associated formulas are described below and in Supplementary Table S2.

Digital shoot area Equation (1) from the images was calculated using the formula from [83]:

(1) $$\mathrm{Digital}\;\mathrm{shoot}\;\mathrm{area}\;(A\_V_{\mathrm{IAP}})=\sqrt{A_{s.Average}^{2}\times A_{t.Average}}$$

where *A_s.Average_* is the average pixel area from both side angle views and *A_t.average_* is the average pixel area from the top view.

The digital shoot growth rate (dSGR) was calculated from the digital shoot area across time points as per Equation (2) and implemented in R package lme4 [84]:

Y = β0 + gt(2)

where Y is the digital shoot area, β0 is the digital shoot area on the first day after treatment, t is the time period, and g is the shoot growth rate (slope). The slope derived from this model for each accession was used as the digital shoot growth rate.

Salt susceptibility index (SSI) Equation (3) for each of the traits was calculated as an index between the control and treatment plants based on the modified formula from [85]:

(3) $$\mathrm{SSI}=(1-\frac{T_{t}}{T_{c}})/(1-\frac{\mu_{t}}{\mu_{c}})$$

where *T_t_* is the trait value under treatment (125 mM NaCl), *T_c_* is the trait value under control (0 mM NaCl), *µ_t_* is the whole population mean of the trait under treatment, and *µ_c_* is the whole population mean of the trait under control conditions.

The digital senescence rate (dSR) was calculated with Equation (2) using yellow and necrotic pixel areas for each plant across all timepoints.

All the correlation values mentioned as ‘r’ in the manuscript were analysed as Pearson’s correlation coefficient values.

### 4.4. Ion Analysis of Leaf

For Na^+^ and K^+^ analysis, leaves were digested in 1% (*v*/*v*) nitric acid at 100 °C for 4 h in a water bath. The Na^+^ and K^+^ concentrations of the digested leaf material were determined using a flame photometer (Sherwood 420, Sherwood Scientific, Cambridge, UK), following the protocol described in [86].

### 4.5. Genotype Data Analysis and Haplotype Blocks

Exome SNP genotype calls reported in He et al. [47] for the AMP accessions were filtered to remove SNPs with <60% call rate, <1% MAF, and high (>5%) heterozygosity. After filtering, a set of 510 accessions with 883,300 SNPs was used to define haplotype blocks (hapblocks) following the method described by [87] and implemented in Plink V1.9 [88] based on the confidence interval of pairwise LD between SNPs within a maximum 1 MB window. Genomic regions in which >95% of SNP pair comparisons showed strong LD were considered a single haplotype block. Regions where LD extended beyond 1 MB were reported as multiple haplotype blocks to allow extremely large blocks to be avoided and keep the resolution of mapping to a 1 MB interval where possible. The SNPs within the defined hapblocks were used to identify hapalleles and to construct a hapallele matrix (Hap-matrix) using the Ghap R-package [89]. The genotype scoring approach in Ghap follows additive SNP coding, wherein each hapallele is considered a pseudo-marker and is written to a new column with scores 0, 1, and 2 corresponding to zero, one, or two copies of the allele, respectively. The midpoint between the start and end of the hapblock is considered the genomic position of each hapallele in the Hap-matrix. Hapblock statistics were calculated using the Ghap R-package, and low-frequency hapalleles (MAF < 1%) were excluded from further analysis. The final Hap-matrix consisted of unique hapalleles (with MAF > 1%) in individual columns with corresponding scores and genetic positions.

### 4.6. Haplotype-Based GWAS (HGWAS)

A set of 102,159 LD-pruned SNPs derived by filtering out SNPs within a window size of 5MB with an LD threshold r^2^ > 0.2 were used to perform PCA and kinship matrix calculation (Plink V1.9; [88]). HGWAS was performed for each trait using a MLM model with the first four principal components as fixed effects, the kinship matrix as random effects, and the Hap-matrix as genotypes. Genomic heritabilities were calculated for each trait using the genomic relationship matrix (GRM) estimated from the Hap-matrix using GREML in GCTA [90]. Traits with less than 0.2 genomic heritability were excluded from HGWAS analysis. Genetic correlations describing the shared genetic architecture between traits were estimated in MTG2 [91] with the Hap-matrix.

### 4.7. Significant Marker Trait Associations and Cross-Validation of HGWAS

A K-fold cross-validation approach was used to confirm the significance of hapallele trait associations. The AMP accessions were randomly divided into five equal-sized groups, and HGWAS analysis was performed using four of the groups (80% of samples), with the fifth group left out each time. For each trait, five-fold cross-validation was run 20 times, amounting to 100 runs per trait. Hapallele trait associations with a log_10_ *p*-value > 5.00 (*p*-value < 0.00001) in more than 50 of the 100 cross-validation runs were considered to be significantly associated with the trait. The modified Bonferroni correction method proposed for GWAS studies [92,93], which uses the number of LD-based haplotype blocks as the effective number of independent tests (n_e_), was used to correct for multiple testing. Using this method, the corrected significance *p*-value threshold was determined as α divided by the number of LD-based haplotype blocks (0.05/58502), resulting in a *p*-value significance threshold of 8.54 × 10^−7^.

### 4.8. Gene-Based Meta-Analysis

Gene-based meta-analysis was performed using FastBAT [94] implemented in GCTA [90] for genes within the delineated QTL regions. FastBAT is a gene-level test approach that uses SNP association summary results from GWAS and LD measures between the SNPs in each gene to provide gene-based estimates for the significance of association. Coordinates from the IWGSC RefSeq v2.0 [42] genome assembly for cultivar Chinese Spring were used to define the intervals for genes underlying the QTL. FastBAT was used to calculate the gene-level association *p*-value for the input list of genes by considering all the SNP *p*-values within 2 kb of the gene and the LD r^2^ values between the SNPs from individual genotype data. A meta-analysis was performed for each trait separately, and genes with a trait association *p*-value < 0.001 were considered significant.

Gene ontology enrichment analysis was performed using PANTHER [95] and agriGO v2.0 [96] for the full list of genes underlying the QTL and separately for the genes found to be significant in the GWAS meta-analysis. The single enrichment analysis method implemented in agriGO v2.0 was used for GO term enrichment analysis, wherein the list of genes tested was compared with a reference set of genes to calculate time enrichment, and the Fisher test was used to determine significance. Only GO terms significant at a *p*-value of 0.05 were reported and discussed (Figure 6). Full gene lists, GO terms, their fold enrichment, and Fisher test *p*-values are reported in Supplementary Table S6, and the gene lists significant in gene-based meta-analysis are reported in Supplementary Table S7. GO term overrepresentation analysis was also performed in PANTHER and compared to the agriGO results. Results from PANTHER were used to compare the top-enriched GO terms in both approaches.

## Data Availability

The datasets supporting the results of this article are included within the article.

## Supplementary Materials

The following supporting information can be downloaded at: https://www.mdpi.com/article/10.3390/plants12122367/s1, Supplementary Figure S1. Correlation between measured dry biomass and total pixel area at last timepoint of imaging. *Y*-axis and *x*-axis show digital shoot are and manual harvested dry biomass. Green circles: control treatment; Red triangles: salt treatment. Supplementary Figure S2. Above ground biomass and derived plant growth rate measured using HTP-imaging platform in PPV-B. (A) Total pixel area which correlates to biomass was measured every alternate day on both the control and salt treated plants. (B) The slopes from the plant growth model including all the time-point data were used as plant growth rate for each accession. *Y*-axis shows the total pixel area, *x*-axis shows the imaging time point (days after treatment) and are colour coded for treatment (Green: control; Red: salt). Supplementary Figure S3. Sodium (Na^+^) accumulation in youngest shoot tissue (2nd leaf from top). The top panel of plots corresponds to Na+ in the PPV-B and bottom panel of plots for the PPV-H. *Y*-axis corresponds to Na+ values in 2nd leaf. *p*-value for the test of significant difference between means of control and salt treatments based on ANOVA is shown on top left of the figure (*** denotes *p*-value < 0.0001). Colour code: Red- Tolerant in both locations; Yellow- Susceptible in both locations; Grey-Moderately tolerant to susceptible. (A) Histogram showing distribution of Na+ concentrations in leaf tissue under both control and salt treated conditions. (B) Boxplots showing the phenotype variation. (C) Reaction-norm plots for Na^+^ between control and salt treated plants. Supplementary Figure S4. Potassium (K^+^) accumulation in the youngest shoot tissue (2^nd^ leaf from top). The top panel of plots corresponds to K^+^ in the PPV-B and bottom panel of plots for the PPV-H. *Y*-axis corresponds to K^+^ accumulation values in young leaf tissue. *p*-value for the test of significant difference between means of control and salt treatments based on ANOVA is shown on top left of the figure (*** denotes *p*-value < 0.0001). Colour code: Red- Tolerant in both locations; Yellow- Susceptible in both locations; Grey- Moderately tolerant to susceptible. A) Histogram showing distribution of K^+^ concentrations in leaf tissue under both control and salt treated conditions. B) Boxplots showing the phenotype variation. C) Reaction-norm plots for K^+^ between control and salt treated plants. Supplementary Figure S5. Potassium (K^+^) accumulation in the oldest shoot tissue (4th leaf from bottom). The top panel of plots corresponds to K^+^ in the PPV-B and bottom panel of plots for the PPV-H. *Y*-axis corresponds to K^+^ values in oldest shoot tissue. *P*-value for the test of significant difference between means of control and salt treatments based on ANOVA is shown on top left of the figure (*** denotes *p*-value < 0.0001). Colour code: Red, tolerant in both locations; Yellow, susceptible in both locations; Grey, moderately tolerant to susceptible. (A) Histogram showing distribution of K^+^ concentrations in leaf tissue under both control and salt treated conditions. (B) Boxplots showing the phenotype variation. (C) Reaction-norm plots for K^+^ between control and salt treated plants. Supplementary Figure S6. Potassium to sodium (K^+^/Na^+^) ratio in oldest shoot tissue (4^th^ leaf from bottom). The top panel of plots corresponds to K^+^/Na^+^ in the PPV-B and bottom panel of plots for the PPV-H. *Y*-axis corresponds to K^+^/ Na^+^ ratio values in oldest leaf tissue. *P*-value for the test of significant difference between means of control and salt treatments based on ANOVA is shown on top left of the figure (*** denotes *p*-value < 0.0001). Colour code: Red, tolerant in both locations; Yellow, susceptible in both locations; Grey, moderately tolerant to susceptible. (A) Histogram showing distribution of K^+^/Na^+^ ratios in leaf tissue under both control and salt treated conditions. (B) Boxplots showing the phenotype variation. (C) Reaction-norm plots for K^+^/Na^+^ between control and salt treated plants. Supplementary Figure S7. SNP distribution and haploblock distribution across chromosomes in wheat collection. Tracks from periphery to centre show haploblocks per MB, average number of hapalleles per MB; Hapalleles per block; average block size per Mb and haploblocks per gene. Supplementary Figure S8. Correlogram of genetic correlations between the salinity tolerance traits measured in both locations. Only genetic correlations that were significant are represented here and blue and red colour intensity represent magnitude of negative and positive values, respectively. Supplementary Figure S9. Gene-based meta-analysis results for genes underlying 96 QTL regions. Colour code: Red, growth related traits; Green, leaf ion concentrations; and Purple, salinity stress index. *X*-axis shows the physical position of genes and the *y*-axis shows log_10_(*p*-value) of the top SNP from the gene. Chromosome number and sub-genome are shown on the plot. Supplementary Figure S10. Gene ontology (GO) enrichment analysis with full list of genes (*n* = 3,134) underlying the salinity tolerance QTL. Only top 20% GO terms among significant GO term enrichment are shown. Colour code: Blue, cellular component GO term; Red, molecular function; and Green, Biological function GO term. Supplementary Figure S11. PCA of the diverse wheat collection with 100,000 SNPs. Resistant lines (red dots) and susceptible lines (blue dots). Supplementary Table S1. List of accessions screened for salinity tolerance. Columns indicate accession name, exome sequenced accessions, domestication status, country of origin, geographical region, and location of salinity screening in PPV-B and PPV-H. Susceptible and tolerant accessions. Supplementary Table S2. Description of salinity tolerance traits measured and calculated. The traits measured in PPV-H and PPV-B are indicated in columns C & D. Supplementary Table S3. List of QTL associated with salinity tolerance component traits. List of QTL their physical positions, associated trait names, and corresponding overlapping QTL reported in previous salinity tolerance studies are provided. Columns indicate QTL name, chromosome, position, trait names, overlap QTL from literature. QTL that did not pass Bonferroni correction P-Value are highlighted in red. Supplementary Table S4. List of Genes underlying 95 QTL regions detected by HGWAS analysis. Column names indicate Gene ID (IWGSc.v2), chromosome, start of the gene, end position of the gene, Blast hit, Gene function, GO-IDs and corresponding QTL name. Gene function, GO-IDs and corresponding QTL name.Gene function description indicating involvement in either sodium or potasssium transport or in ion transport activity are indicated in last two columns. Supplementary Table S5. List of genes significant in gene based meta-analysis using FastBAT method. Column names indicate Gene ID (IWGSC.v2), chromosome, start of the gene, end position of the gene, Blast hit, Gene function, GO-IDs and corresponding QTL name.Gene function description indicating involvement in either sodium or potassium transport or in ion transport activity are indicated in last two columns. Supplementary Table S6. Significant GO terms from gene enrichment analysis of full list of genes under 95 QTL intervals. Term_type indicates P-Biological processes; F-Molecular function and C-Cellular function related GO terms. Supplementary Table S7. Results from gene enrichment analysis of genes (*n* = 107) significant in gene based meta-analysis. Term_type indicates P-Biological processes; F-Molecular function and C-Cellular function related GO terms.
