# Supplementary material for: Haplotype-Based Genome-Wide Association Analysis Using Exome Capture Assay and Digital Phenotyping Identifies Genetic Loci Underlying Salt Tolerance Mechanisms in Wheat
Source: Plants (Basel). 2023 Jun 19;12(12):2367. doi: 10.3390/plants12122367 (PMC10301518; doi:10.3390/plants12122367)
Supplement: Supplementary file 1 [file plants-12-02367-s001.zip › Wheat Salinity_Supplementary Figures.pptx]

## Slide 1
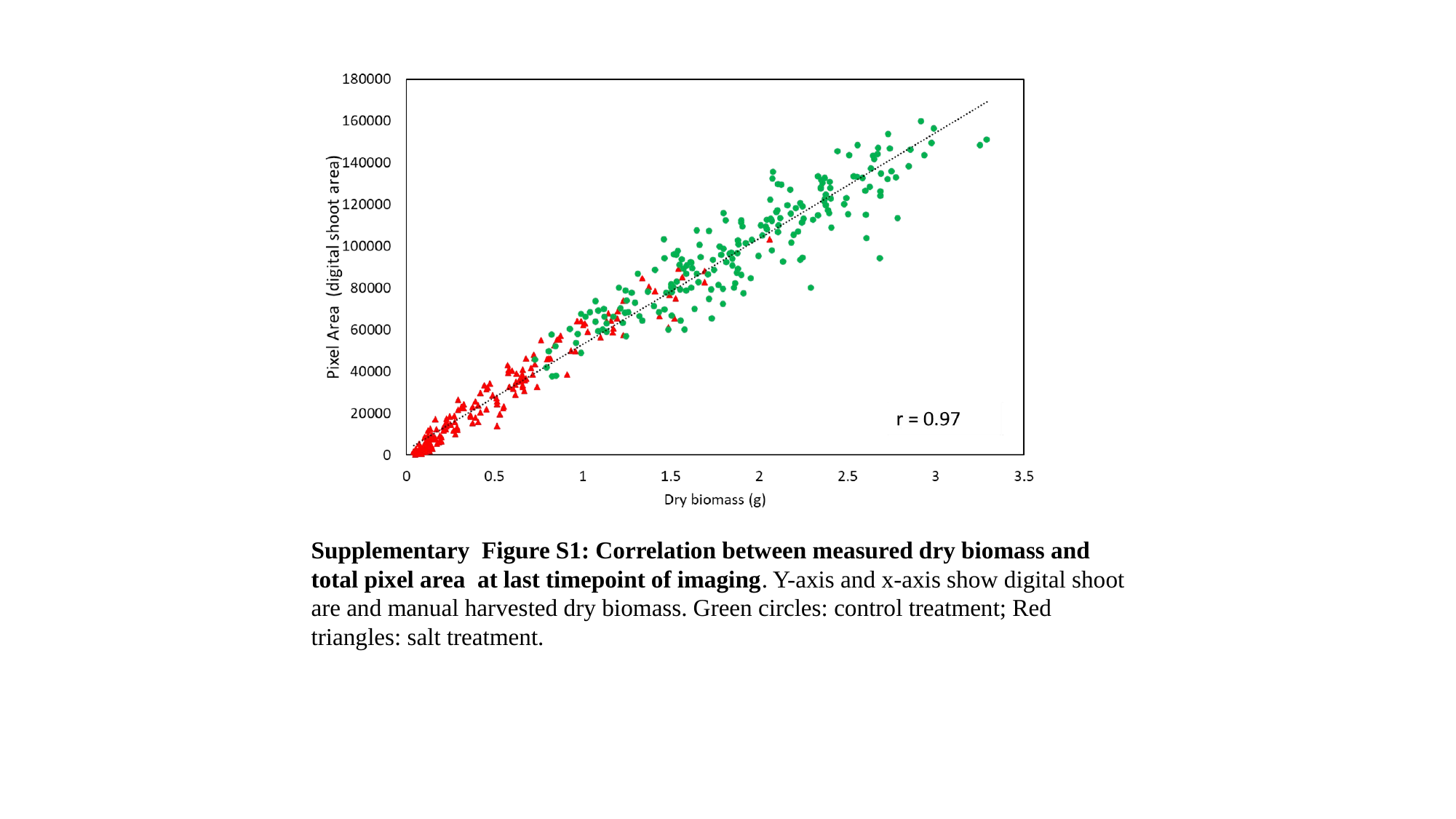

Supplementary Figure S1: Correlation between measured dry biomass and total pixel area at last timepoint of imaging. Y-axis and x-axis show digital shoot are and manual harvested dry biomass. Green circles: control treatment; Red triangles: salt treatment.

## Slide 2
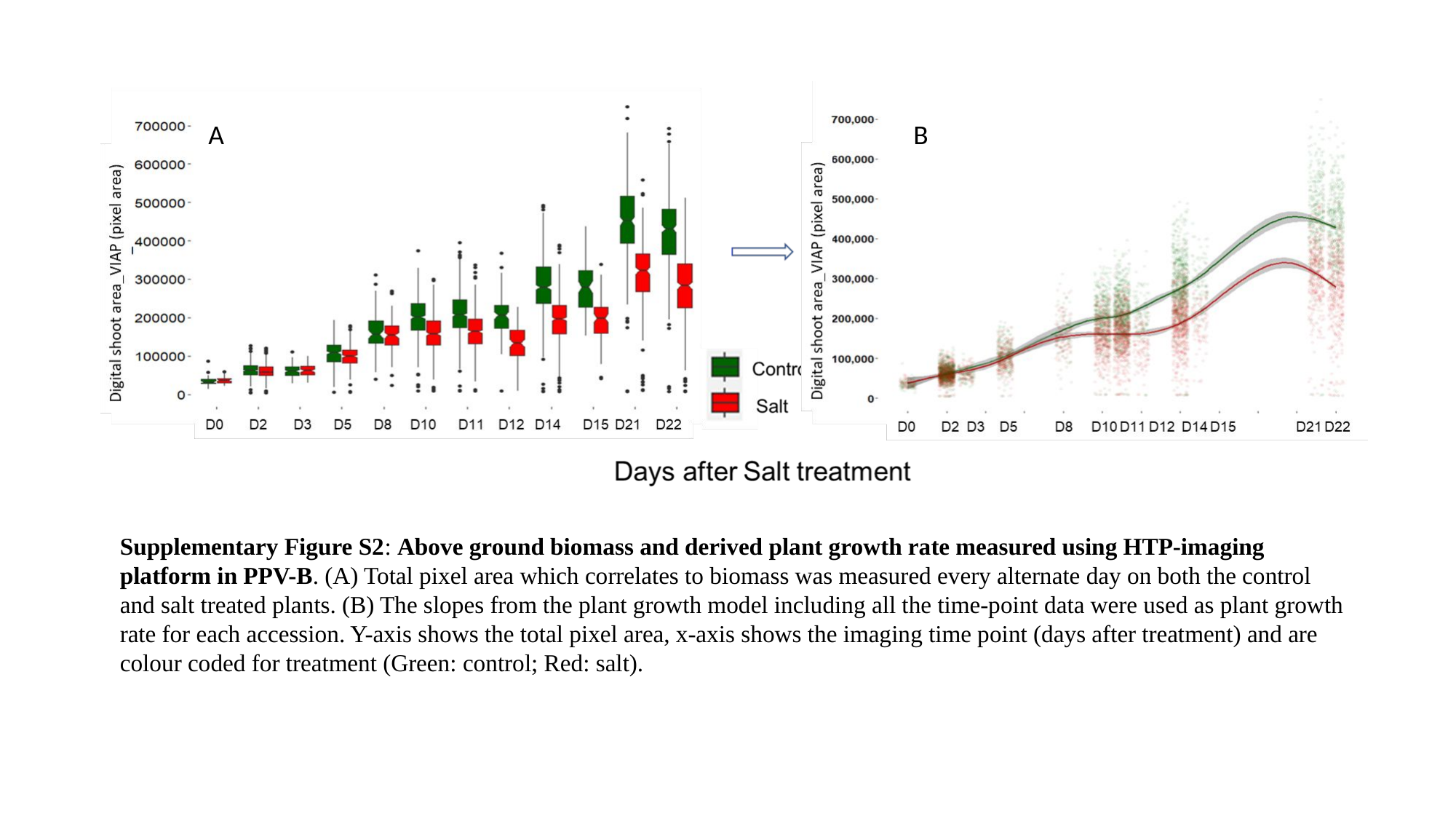

A
B
Supplementary Figure S2: Above ground biomass and derived plant growth rate measured using HTP-imaging platform in PPV-B. (A) Total pixel area which correlates to biomass was measured every alternate day on both the control and salt treated plants. (B) The slopes from the plant growth model including all the time-point data were used as plant growth rate for each accession. Y-axis shows the total pixel area, x-axis shows the imaging time point (days after treatment) and are colour coded for treatment (Green: control; Red: salt).

## Slide 3
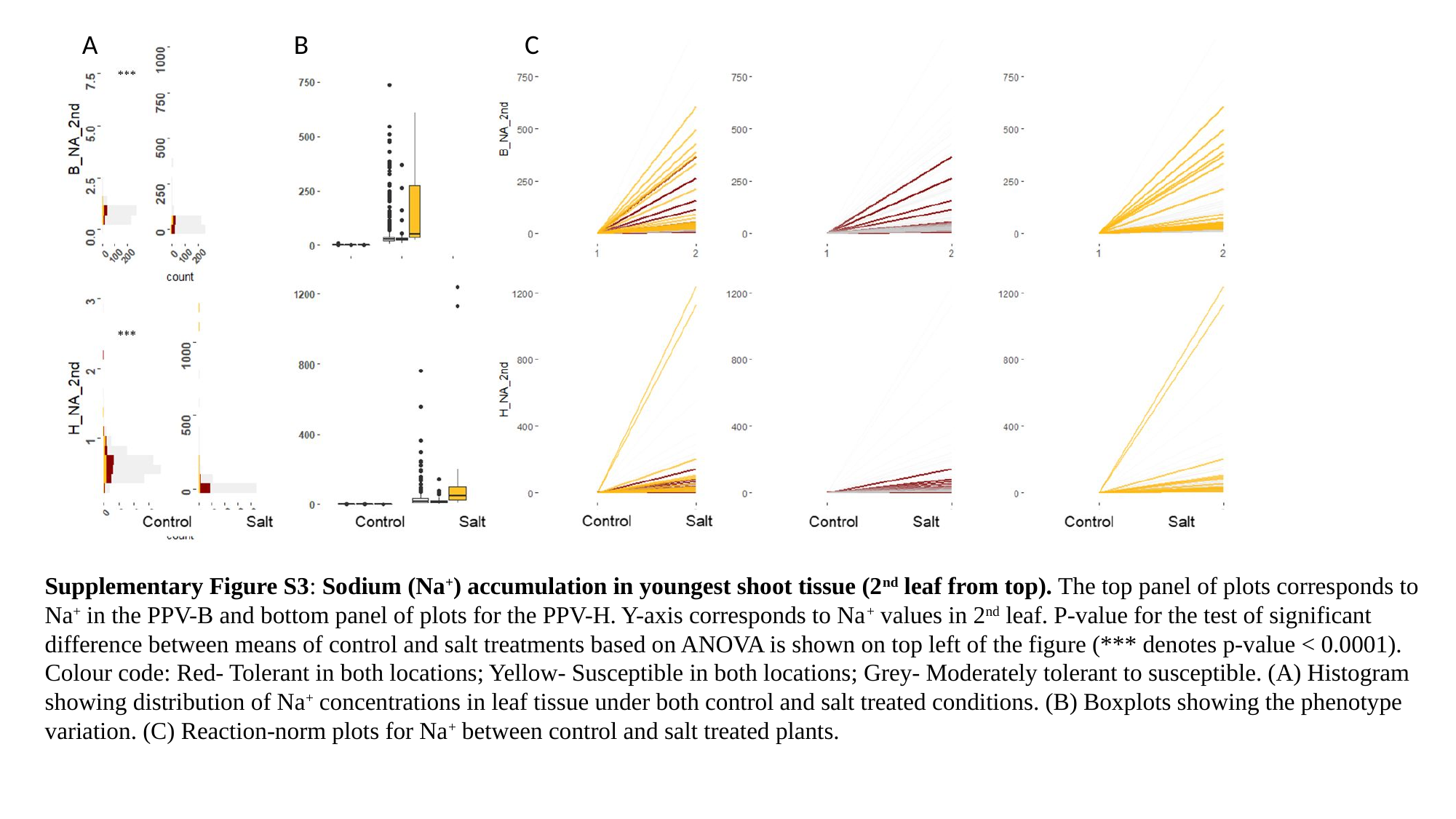

A
B
C
Supplementary Figure S3: Sodium (Na+) accumulation in youngest shoot tissue (2nd leaf from top). The top panel of plots corresponds to Na+ in the PPV-B and bottom panel of plots for the PPV-H. Y-axis corresponds to Na+ values in 2nd leaf. P-value for the test of significant difference between means of control and salt treatments based on ANOVA is shown on top left of the figure (*** denotes p-value < 0.0001). Colour code: Red- Tolerant in both locations; Yellow- Susceptible in both locations; Grey- Moderately tolerant to susceptible. (A) Histogram showing distribution of Na+ concentrations in leaf tissue under both control and salt treated conditions. (B) Boxplots showing the phenotype variation. (C) Reaction-norm plots for Na+ between control and salt treated plants.

## Slide 4
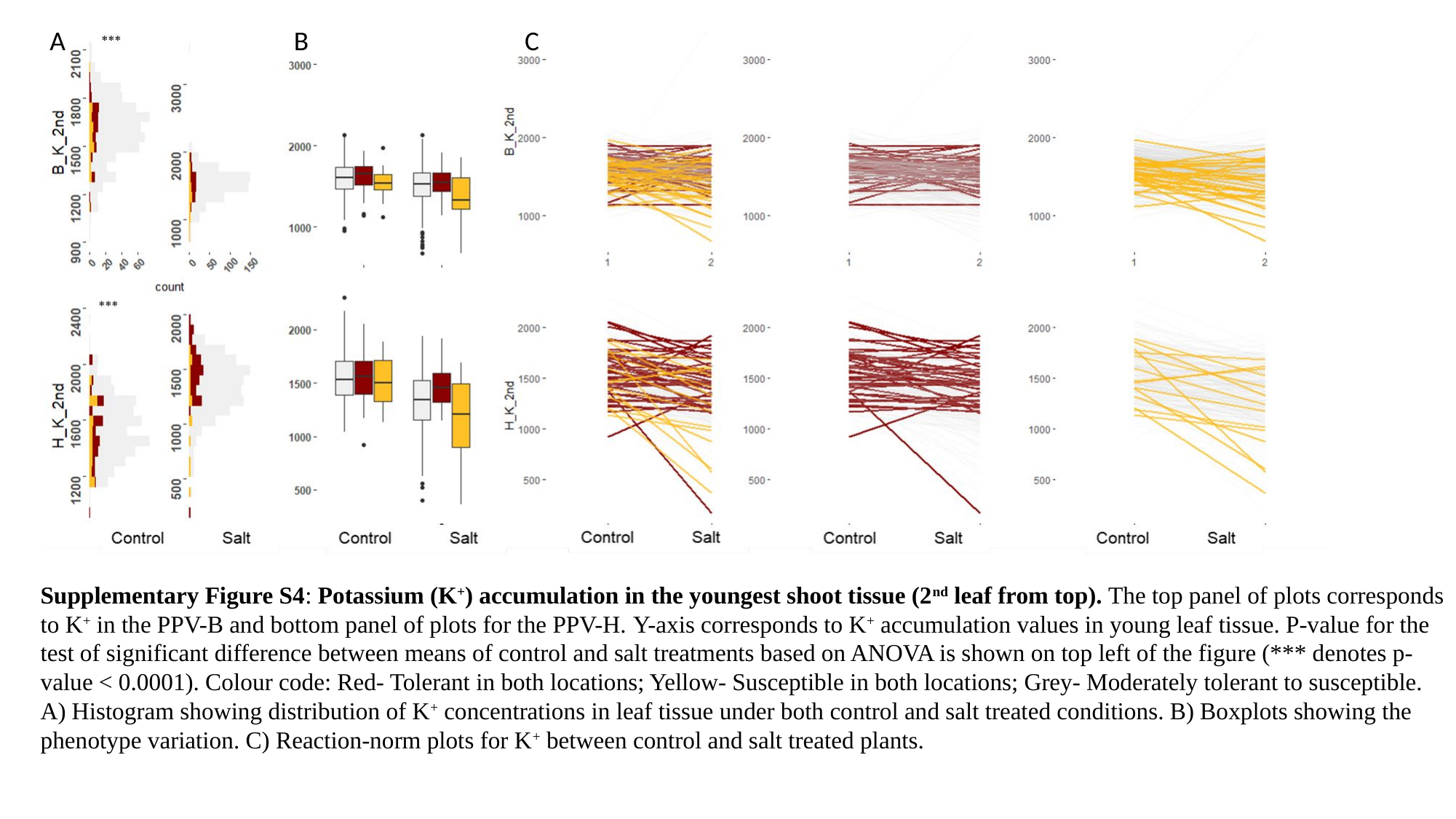

A
B
C
Supplementary Figure S4: Potassium (K+) accumulation in the youngest shoot tissue (2nd leaf from top). The top panel of plots corresponds to K+ in the PPV-B and bottom panel of plots for the PPV-H. Y-axis corresponds to K+ accumulation values in young leaf tissue. P-value for the test of significant difference between means of control and salt treatments based on ANOVA is shown on top left of the figure (*** denotes p-value < 0.0001). Colour code: Red- Tolerant in both locations; Yellow- Susceptible in both locations; Grey- Moderately tolerant to susceptible. A) Histogram showing distribution of K+ concentrations in leaf tissue under both control and salt treated conditions. B) Boxplots showing the phenotype variation. C) Reaction-norm plots for K+ between control and salt treated plants.

## Slide 5
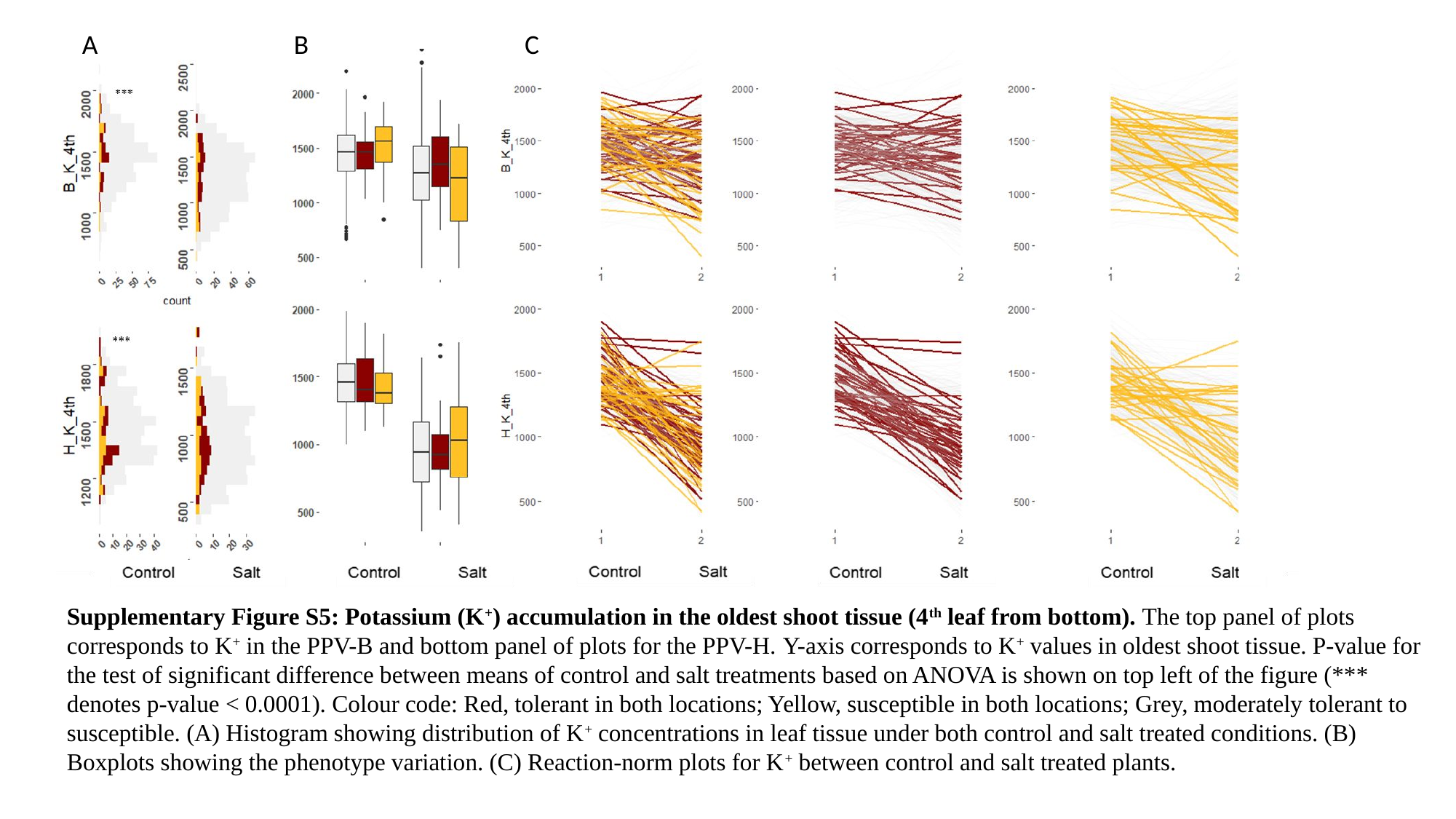

A
B
C
Supplementary Figure S5: Potassium (K+) accumulation in the oldest shoot tissue (4th leaf from bottom). The top panel of plots corresponds to K+ in the PPV-B and bottom panel of plots for the PPV-H. Y-axis corresponds to K+ values in oldest shoot tissue. P-value for the test of significant difference between means of control and salt treatments based on ANOVA is shown on top left of the figure (*** denotes p-value < 0.0001). Colour code: Red, tolerant in both locations; Yellow, susceptible in both locations; Grey, moderately tolerant to susceptible. (A) Histogram showing distribution of K+ concentrations in leaf tissue under both control and salt treated conditions. (B) Boxplots showing the phenotype variation. (C) Reaction-norm plots for K+ between control and salt treated plants.

## Slide 6
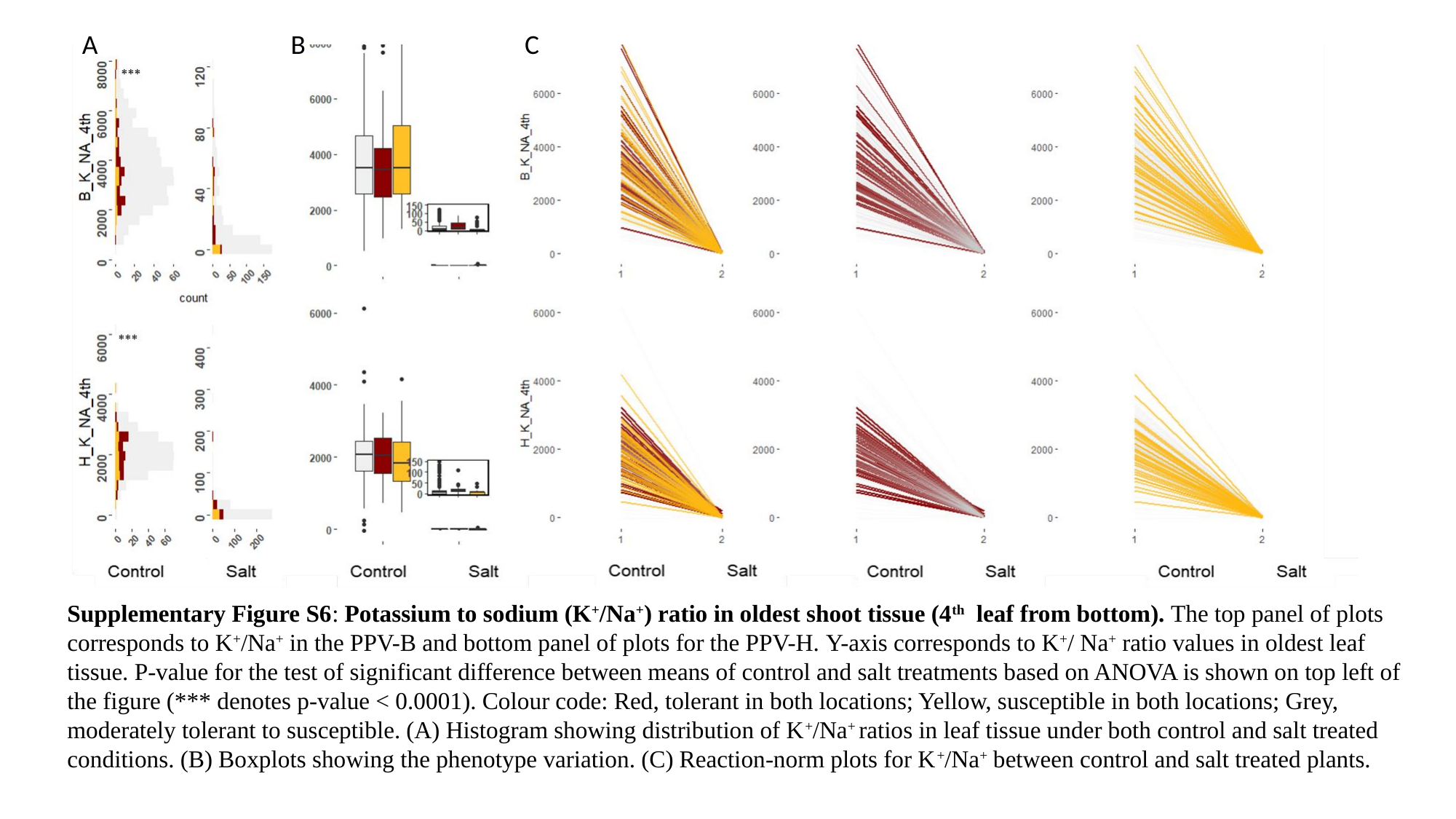

A
B
C
Supplementary Figure S6: Potassium to sodium (K+/Na+) ratio in oldest shoot tissue (4th leaf from bottom). The top panel of plots corresponds to K+/Na+ in the PPV-B and bottom panel of plots for the PPV-H. Y-axis corresponds to K+/ Na+ ratio values in oldest leaf tissue. P-value for the test of significant difference between means of control and salt treatments based on ANOVA is shown on top left of the figure (*** denotes p-value < 0.0001). Colour code: Red, tolerant in both locations; Yellow, susceptible in both locations; Grey, moderately tolerant to susceptible. (A) Histogram showing distribution of K+/Na+ ratios in leaf tissue under both control and salt treated conditions. (B) Boxplots showing the phenotype variation. (C) Reaction-norm plots for K+/Na+ between control and salt treated plants.

## Slide 7
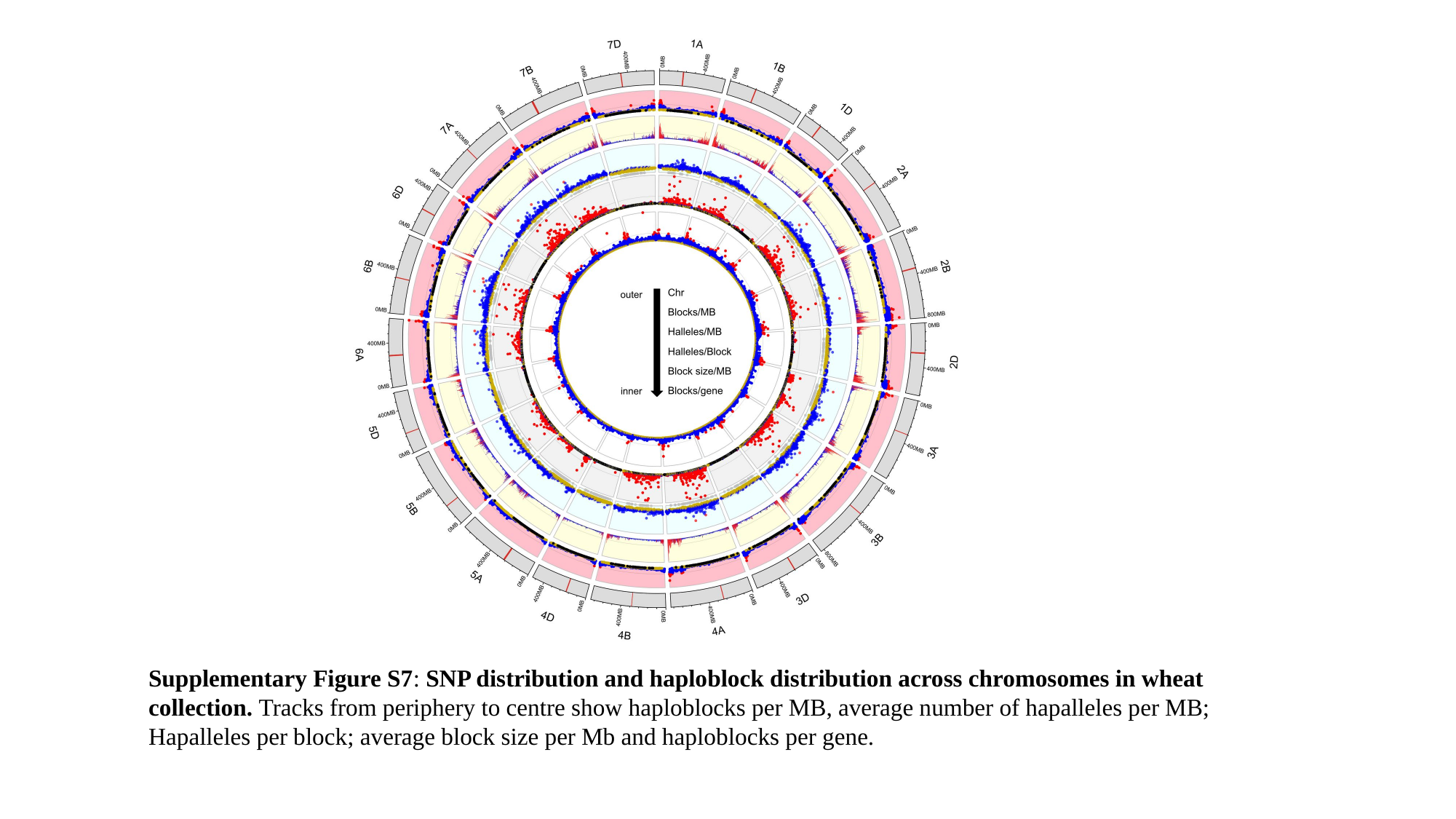

Supplementary Figure S7: SNP distribution and haploblock distribution across chromosomes in wheat collection. Tracks from periphery to centre show haploblocks per MB, average number of hapalleles per MB; Hapalleles per block; average block size per Mb and haploblocks per gene.

## Slide 8
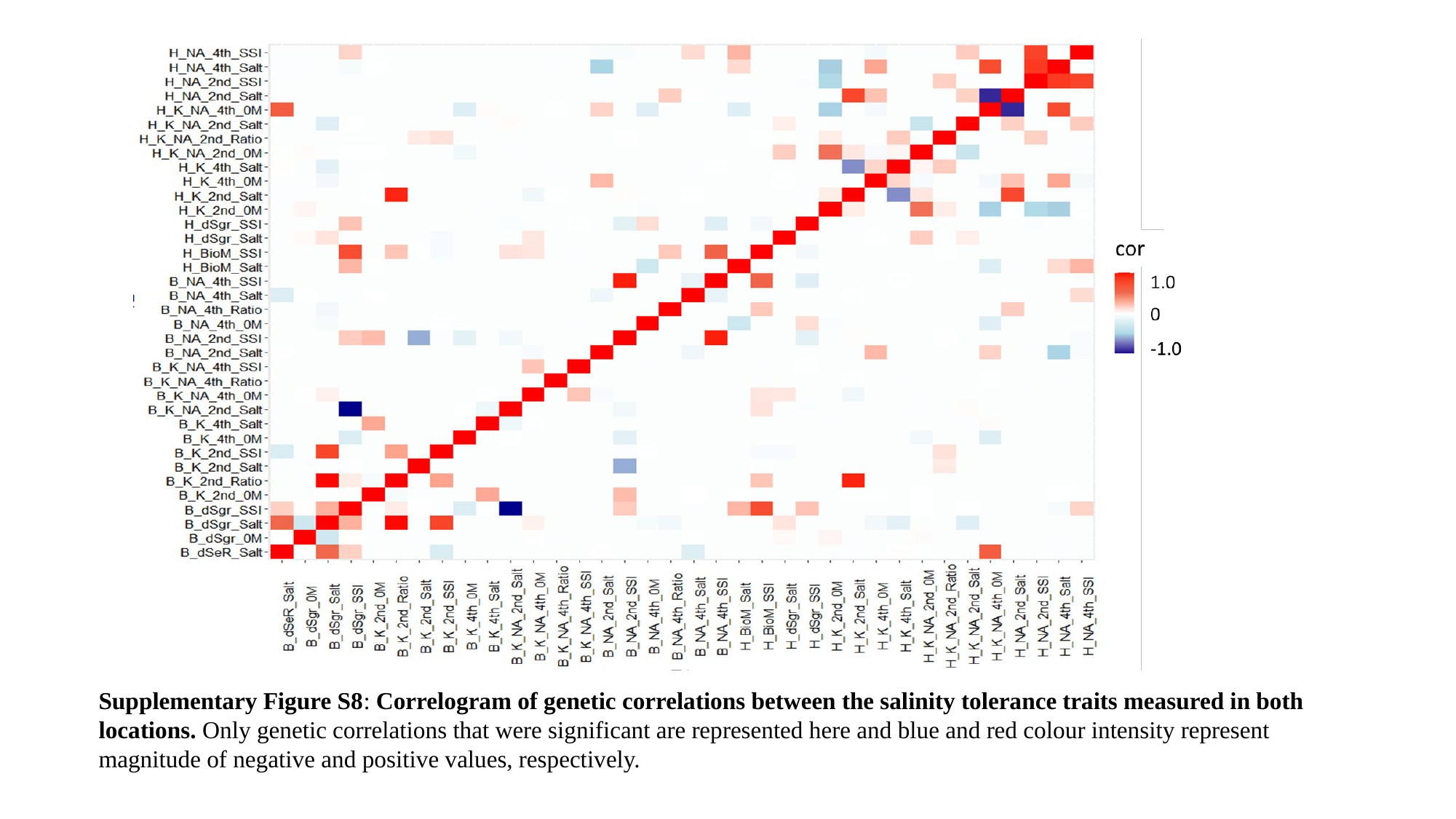

Supplementary Figure S8: Correlogram of genetic correlations between the salinity tolerance traits measured in both locations. Only genetic correlations that were significant are represented here and blue and red colour intensity represent magnitude of negative and positive values, respectively.

## Slide 9
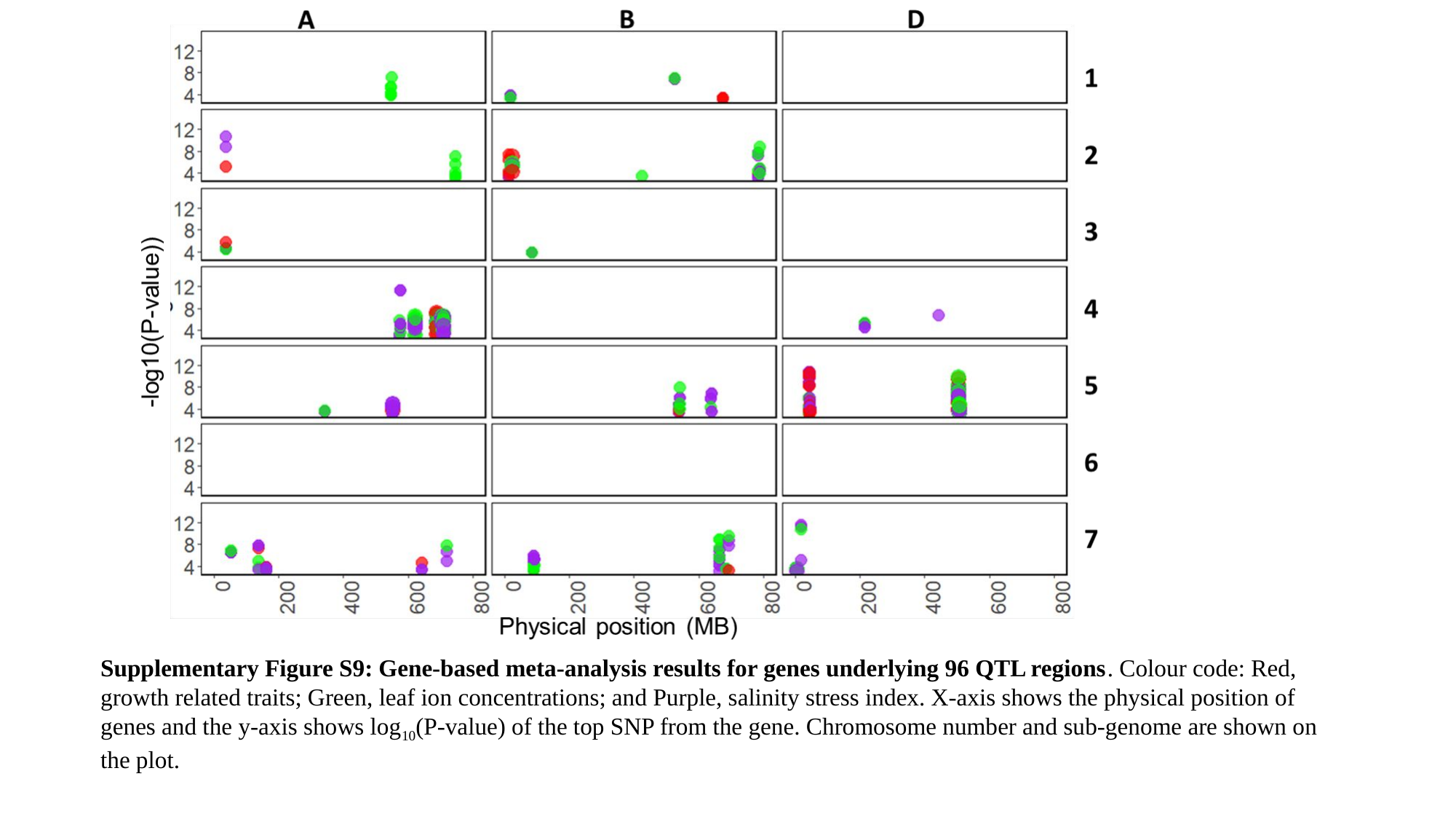

Supplementary Figure S9: Gene-based meta-analysis results for genes underlying 96 QTL regions. Colour code: Red, growth related traits; Green, leaf ion concentrations; and Purple, salinity stress index. X-axis shows the physical position of genes and the y-axis shows log10(P-value) of the top SNP from the gene. Chromosome number and sub-genome are shown on the plot.

## Slide 10
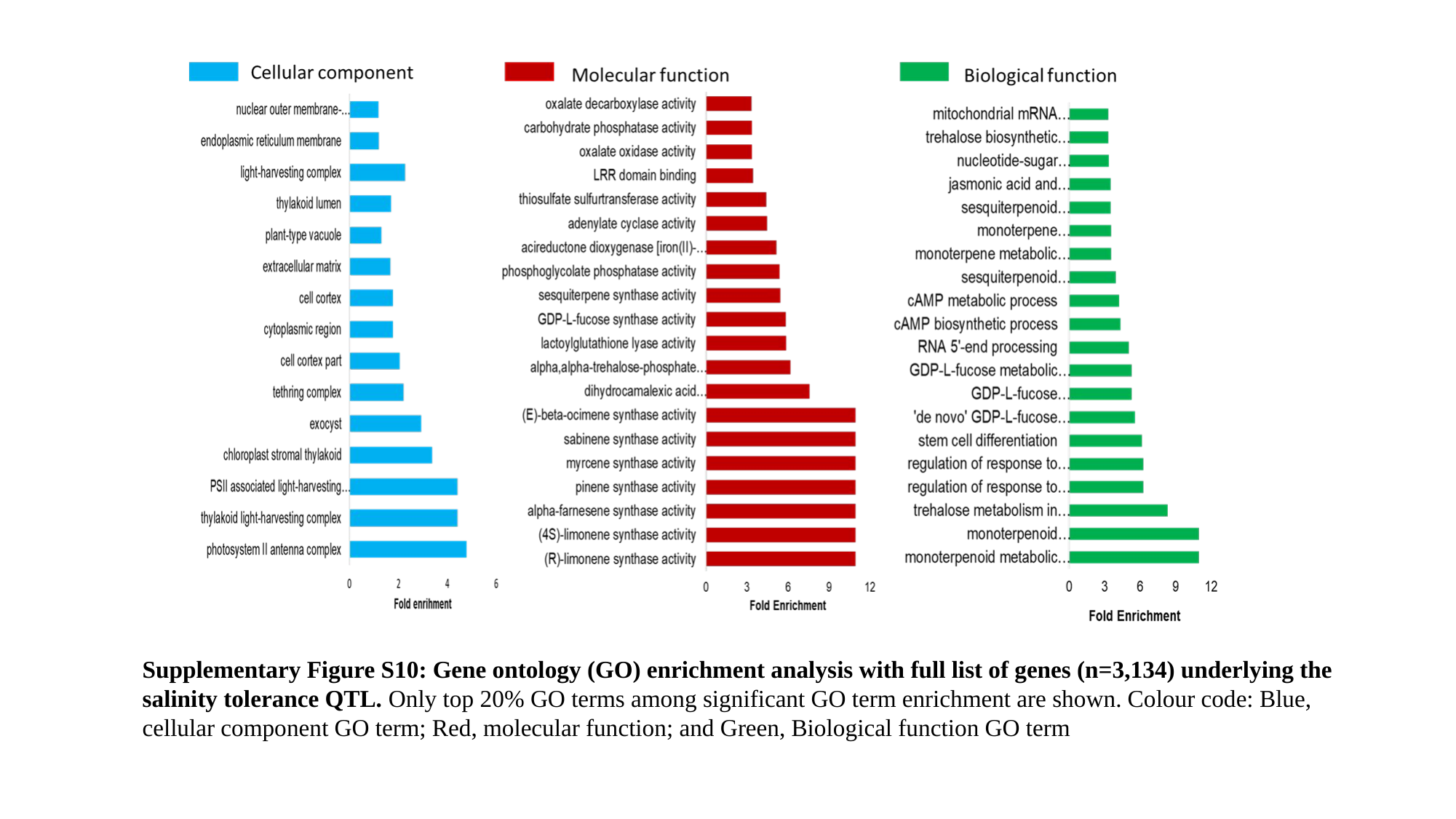

Supplementary Figure S10: Gene ontology (GO) enrichment analysis with full list of genes (n=3,134) underlying the salinity tolerance QTL. Only top 20% GO terms among significant GO term enrichment are shown. Colour code: Blue, cellular component GO term; Red, molecular function; and Green, Biological function GO term

## Slide 11
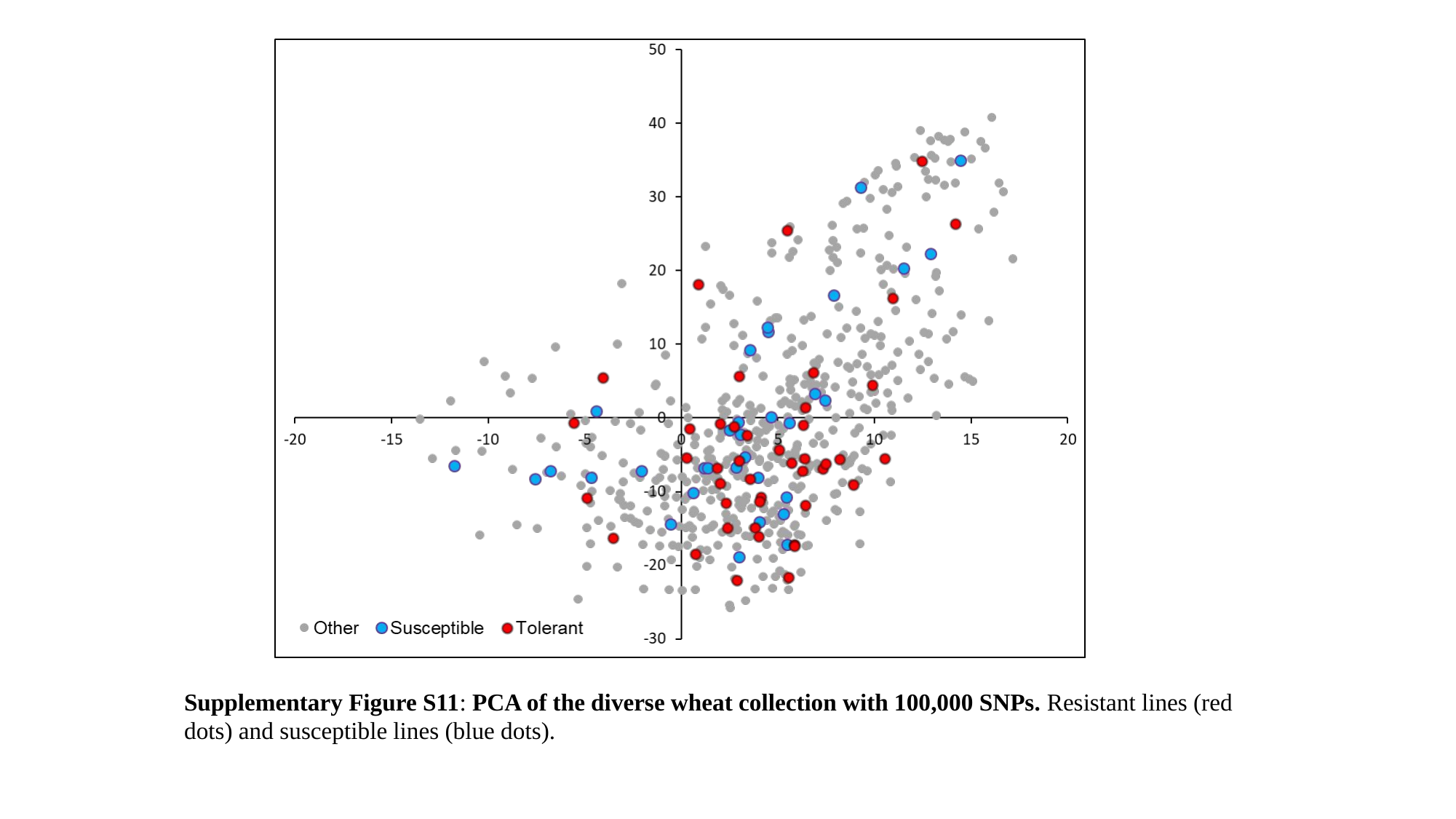

Supplementary Figure S11: PCA of the diverse wheat collection with 100,000 SNPs. Resistant lines (red dots) and susceptible lines (blue dots).
